# Coding of Velocity Storage in the Vestibular Nuclei

**DOI:** 10.3389/fneur.2017.00386

**Published:** 2017-08-16

**Authors:** Sergei B. Yakushin, Theodore Raphan, Bernard Cohen

**Affiliations:** ^1^Department of Neurology, Icahn School of Medicine at Mount Sinai, New York, NY, United States; ^2^Department of Computer and Information Science, Brooklyn College (CUNY), Brooklyn, NY, United States

**Keywords:** monkey, vestibule–ocular reflex, adaptation, gravity, velocity storage, spatial orientation, optokinetic after-nystagmus, vestibular-only neurons

## Abstract

Semicircular canal afferents sense angular acceleration and output angular velocity with a short time constant of ≈4.5 s. This output is prolonged by a central integrative network, velocity storage that lengthens the time constants of eye velocity. This mechanism utilizes canal, otolith, and visual (optokinetic) information to align the axis of eye velocity toward the spatial vertical when head orientation is off-vertical axis. Previous studies indicated that vestibular-only (VO) and vestibular-pause-saccade (VPS) neurons located in the medial and superior vestibular nucleus could code all aspects of velocity storage. A recently developed technique enabled prolonged recording while animals were rotated and received optokinetic stimulation about a spatial vertical axis while upright, side-down, prone, and supine. Firing rates of 33 VO and 8 VPS neurons were studied in alert cynomolgus monkeys. Majority VO neurons were closely correlated with the horizontal component of velocity storage in head coordinates, regardless of head orientation in space. Approximately, half of all tested neurons (46%) code horizontal component of velocity in head coordinates, while the other half (54%) changed their firing rates as the head was oriented relative to the spatial vertical, coding the horizontal component of eye velocity in spatial coordinates. Some VO neurons only coded the cross-coupled pitch or roll components that move the axis of eye rotation toward the spatial vertical. Sixty-five percent of these VO and VPS neurons were more sensitive to rotation in one direction (predominantly contralateral), providing directional orientation for the subset of VO neurons on either side of the brainstem. This indicates that the three-dimensional velocity storage integrator is composed of directional subsets of neurons that are likely to be the bases for the spatial characteristics of velocity storage. Most VPS neurons ceased firing during drowsiness, but the firing rates of VO neurons were unaffected by states of alertness and declined with the time constant of velocity storage. Thus, the VO neurons are the prime components of the mechanism of coding for velocity storage, whereas the VPS neurons are likely to provide the path from the vestibular to the oculomotor system for the VO neurons.

## Definitions

Eigenvector, are orientation vectors associated with the velocity storage system matrix that are activated by stimulus velocity along a specific direction; velocity storage, integrative network of GABA_b_ sensitive neurons in the medial and superior vestibular nucleus (SVN). Type I neurons, neurons receiving convergent input from lateral, anterior, or posterior canals and increase their firing rate with rotation toward that canal located on ipsilateral side of the head. Type II vestibular-only (VO) neurons increase their firing rate with rotation toward the canal located on contralateral side of the head.

## Introduction

When subjects are rotated, the hair cells in the semicircular canals respond to angular acceleration. Because of the elasticity of the cupula and the endolymph a signal related to head velocity is generated in the cupula afferents that decays with a time constant of 3–5 s (1). The sense of rotation, the neural activity in the vestibular nuclei, and the nystagmus generated by a step in head velocity rotation has a time constant of at least 15–25 s, indicating that there is central vestibular processing that lengthens the response time (2–4). The neural mechanism that converts the small time constant response at the canal afferents to the long time constant response found at the level of the vestibular nuclei, has been termed “a velocity storage integrator” (5–7). Full field rotation in light also activates velocity storage probably through the subcortical visual system (8–10). Rotation in light activates velocity storage from both of these modalities so that as the vestibular drive from the semicircular canals wanes, the visual system continues to drive velocity storage generating optokinetic nystagmus (OKN) (6, 8). Moreover, when the lights are extinguished, the optokinetic after-nystagmus (OKAN) has a time constant similar to that induced in darkness by rotation at the same velocity (6, 8, 10).

A key feature of velocity storage is that it is sensitive to the orientation of the head relative to gravity and can be associated with orientation vectors of the velocity storage system matrix that change as a function of head orientation (11–14). If the head is tilted side-down or in forward-back planes, the yaw velocity storage orientation vector shifts so that it tends to align with the spatial vertical (gravity), inducing corresponding pitch or roll slow phase eye velocities in head coordinates (11–14). This cross-coupling of the eye velocity occurs from yaw-to-pitch or roll based on otolith clues but not from roll-pitch to yaw [see Ref. (15, 16) for details].

Neurons that code the slow phase eye velocities of rotational nystagmus and OKN are located in the vestibular nuclei (4, 17, 18), and electric stimulation in this area elicits activation of the velocity storage mechanism (19). Midline sections of the commissural pathways at the level of the rostral medial vestibular nucleus (MVN) and SVN eliminate velocity storage (20, 21). This suggests that the network of neurons in the SVN and MVN and their commissural interconnections are critical for producing velocity storage. A critical finding was that following midline lesions, the degenerated neurons were located in the MVN and SVN and were GABA_b_-ergic (22–24). This raised the possibility that the neurons chiefly responsible for production of velocity storage were a GABA_b_ population. Support for this came from dosage-dependent suppression of velocity storage by intramuscular injection of a GABA_b_ antagonist, baclofen (25, 26).

Using a wide range of stimuli that are known to activate velocity storage indicated that these neurons were VO and vestibular-pause-saccades (VPS) neurons (3). From this, it was hypothesized that the network of neurons in this area that implement velocity storage is presumably comprised of Type I and Type II VO neurons and VPS neurons are part of a later stage in the processing of vestibular signals, carrying the activity from the VPS neurons to the oculomotor system (3). Despite the progress in defining the behavioral properties of velocity storage, however, there has only been sparse information about its neuronal implementation in three dimensions, and those studies that have focused on the neuronal basis of velocity storage have been confined to one dimension, i.e., rotations about the spatial vertical axis (3, 27). These studies have shown that VO and VPS neurons code signals related to velocity storage, but due to the technical difficulties of recording neuronal firing rates stably in various head orientations for long periods of time, the role of the VO neurons in generating the three-dimensional spatial properties of velocity storage remains unexplored.

Novel techniques for long-term recording of neurons in the vestibular nuclei (28–32) have enabled the study of how VO and VPS neurons code the spatial properties of velocity storage in various head orientations. The importance of this is that it would identify the classes of VO and VPS neurons in the SVN and MVN that participate in the spatial orientation of eye velocity. This will fill a gap in our understanding of the importance of the role of VO and VPS neurons in vestibulo-oculomotor processing. Since the VO and VPS neurons are under control of the cerebellar nodulus (33), it should be possible to understand the circuitry in the central vestibular system that orients to the gravitational vertical and gives us a better idea of the underlying cause for the failure to do so.

Some of the data were previously published in abstract form (34). Convergent canal and otolith inputs for neurons in this study were previously published (32). Data on VOR time constant from one neuron in this study before and after baclofen injection were publisher elsewhere (25).

### The Theoretical Basis of Velocity Storage

**Figure 1:**
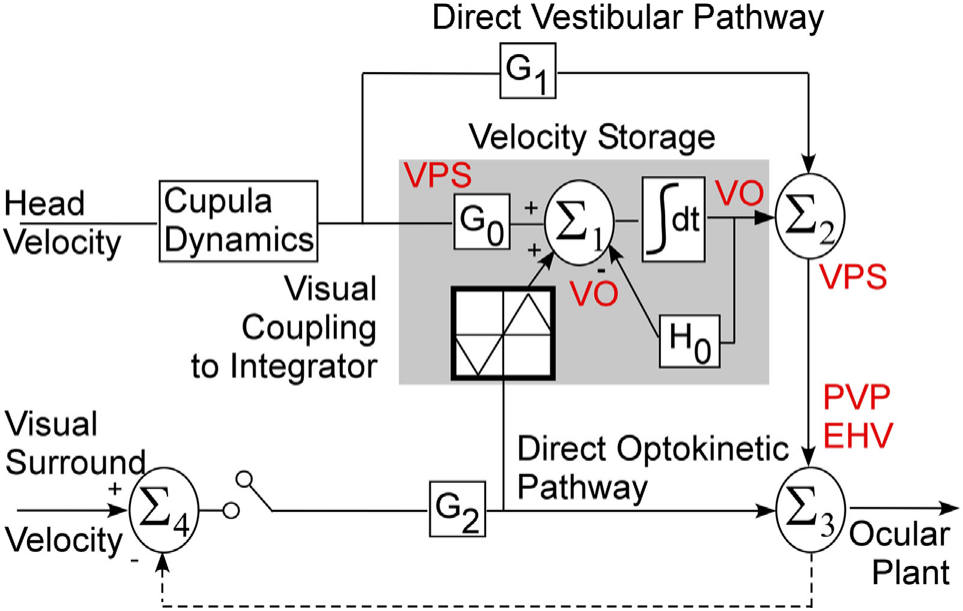
Three-dimensional model of velocity storage integrator. G_0_ represents vestibular input to the integrator (gray area), G_1_ represents direct vestibular, and G_2_ direct optokinetic pathways; H_0_ represents the leak of the integrator. See text for details.

Velocity storage in one dimension was first modeled as a leaky integrator that can be activated by either vestibular or visual inputs. The lengthening of the time constant from that of the vestibular afferent time constant has generated different modeling approaches (6, 7) [see Ref. (5) for a comparison]. Essentially, the time constant is governed by a parameter *h*_0_ in a feedback path whose inverse determines the time constant of the integrator. In three dimensions, the feedback becomes a system matrix, *H*_0_, whose eigenvalues determine the time constants of yaw pitch and roll and whose eigenvectors determine the orientation structure that characterize the orientation (14) (Figure 1). The off-diagonal terms in the system matrix are the cross-coupling parameters and code the spatial orientation aspects of velocity storage (14). This matrix has an upper triangular structure, which reflects the fact that cross-coupling only occurs from yaw-to-roll and -pitch (14). The output signal from the integrator is then summated with the direct vestibular (Σ_1_) and visual (Σ_2_) signals before being processed by the oculomotor system, which includes the velocity-position integrator and plant (35) (Figure 1). Thus, the system matrix determines and predicts both the orientation and temporal properties of response to rotations in tilted head positions and predicts a wide range of experimental outcomes (11–14).

### Relationship of the Model to Central Vestibular Neurons

The Raphan model views the VOR and visual–vestibular interaction as having direct and indirect pathways (Figure 1). Velocity storage is part of the indirect pathway and can be represented as a three-dimensional integrator, characterized by a system matrix (14). In accordance with this model, primary afferents project through a direct vestibular pathway to pre-oculomotor neurons with certain transduction gain level G_1_ (Figure 1). This direct pathway may involve more than just the three-neuron arc (16). The target neurons for this pathway could be position-vestibular-pause (PVP) and/or eye-head-velocity (EHV) neurons (36, 37) or some neurons that directly projects to them as is shown on a schema (Figure 1). The same vestibular afferent activity projects to different central neurons with different transduction gain level G_0_. This signal is summated on neurons that realize the velocity storage network. The velocity storage integrator could be also activated through visual coupling to the integrator (Figure 1). This is accomplished by subtracting eye velocity from velocity of the visual surround, which generates retinal slip and having a dark–light switch, which inactivates the pathway in darkness. During optokinetic following at low frequencies, this pathway mainly activates pre-oculomotor neurons in the vestibular nuclei with a transduction gain G_2_. In contrast to the vestibular input, segregation between direct and indirect optokinetic inputs occurs at the frequency domain of the stimulus. That is, during sinusoidal oscillation of the visual surround at low frequency or during rotation of visual surround at constant velocity this pathway also activates velocity storage (Figure 1). When the light is turned off (open switch) following a period of OKN stimulation, the activity stored in the integrator generates OKAN. In this study, we will relate the recorded neurons to the structure of this three-dimensional model.

## Materials and Methods

The activity of 33 VO and 8 vestibular-pause-saccade (VPS) neurons was recorded in the rostral medial and SVN in three cynomolgus monkeys (*Macaca fascicularis*). First, a head mount was implanted on the skull to provide painless head fixation in stereotaxic coordinates during testing (30, 38). At a second surgery, two three-turn coils were implanted on the left eye. The frontal coil measured the horizontal and vertical components of eye position (39, 40). Another coil, placed on the top of the eye, approximately orthogonal to the frontal coil (41), was used to measure the torsional component of eye position. The surgical procedures were performed under anesthesia in sterile conditions. The surgical procedures and experimental protocol conformed to the Guide for the Care and Use of Laboratory Animals and were approved by the Institutional Animal Care and Use Committee.

### Data Collection and Processing

The animals were tested in a multi-axis vestibular stimulator (3), which has three gimbaled axes for rotation: a horizontal axis parallel to the spatial horizontal, a nested yaw axis, and a doubly nested, inner pitch/roll axis. The yaw and pitch/roll axes were enclosed in a light-tight optokinetic cylinder, 91 cm in diameter, with 10° vertical black and white stripes equally distributed over the visual field. The axis of the cylinder was collinear with the yaw axis. Each axis went through the center of rotation of the head. The animals’ heads were rigidly fixed in the stereotaxic plane.

Voltages related to eye position and to chair rotation about each axis were recorded with amplifiers with a bandpass of DC to 40 Hz, digitized at 500 Hz per channel with a 12-bit resolution, and stored for later analysis. Eye position voltages were smoothed and digitally differentiated by finding the slope of the least squares linear fit, corresponding to a filter with a 3-dB cutoff above 40 Hz, the cutoff frequency of the filters used for data acquisition. Unit activity was converted into pulses (BAK Electronics Inc.) of standard amplitude (5 V) and duration (0.5 ms). Pulses were delayed relative to action potentials by 0.5 ms. The time of the spike occurrence was stored relative to the nearest sampling time with the assumption that only one spike could occur within each sampling period (1.67 ms) or a frequency of 600 Hz (3). Eye movements were calibrated by rotating the animals in light at 30°/s about the pitch, roll, and yaw axes. It was assumed that horizontal and vertical gains were unity and roll gain was ≈0.6 in this condition [see Ref. (42), for details].

Random noise was used to keep animals alert. In some experiments, random noise was not used. Then responses to the same test when animal was and was not drowsy were compared.

### Coordinate Frames

The head coordinate frame was defined by three axes: *x* (naso-occipital, positive direction, back-to-front), *y* (interaural, positive, from the left ear), and *z* (body axis, positive, up). Positive directions for eye movements were defined by the right-hand rule: torsion toward the right ear [clockwise (CW) from the animal’s point of view], vertical down, and left horizontal. Eye movements were defined by the direction of the slow phases of nystagmus, with slow phases to the animal’s right as CW, and *vice versa*, i.e., slow phases to the left as counterclockwise (CCW).

### Unit Recording

**Figure 2:**
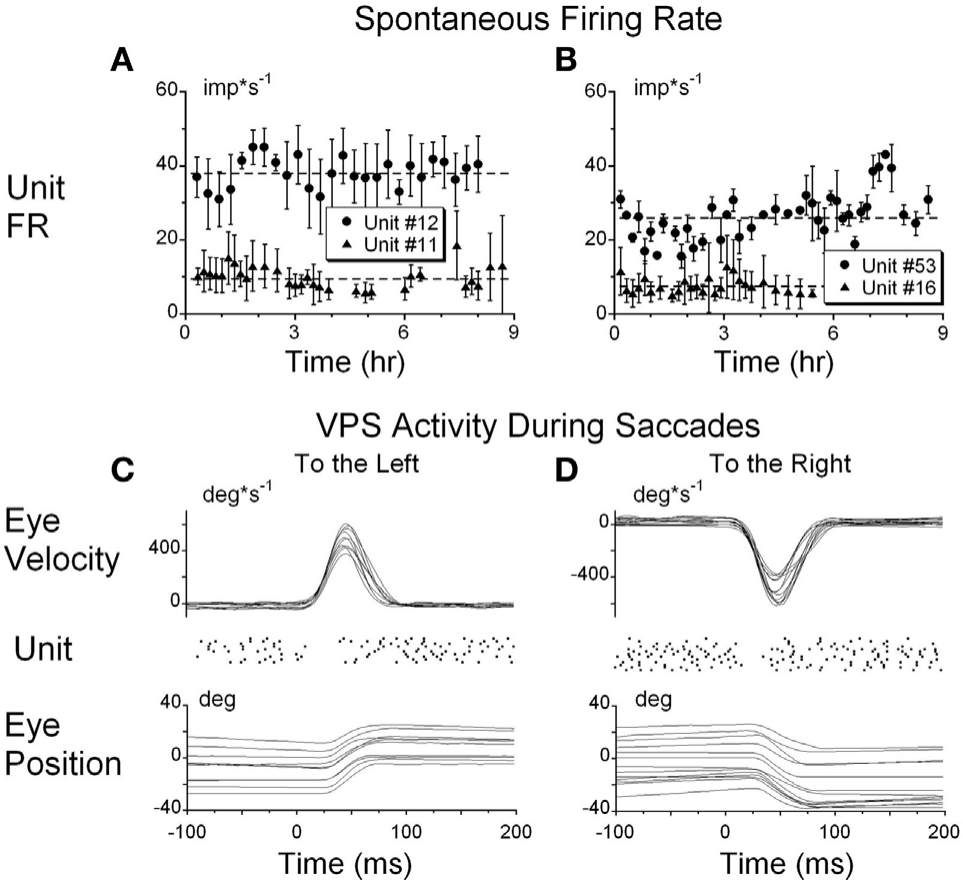
**(A,B)** Typical examples of the resting firing rates from several neurons during the experiments. **(C,D)** Raster’s of firing rate of the position-vestibular-pause (PVP) neuron during spontaneous saccades to the left **(C)** and to the right **(D)**. This PVP neuron paused it firing at the beginning of each saccade.

Details of the unit recording technique are provided elsewhere (29, 32, 38, 43). Briefly, the abducens nucleus was identified first (44) and VO and VPS neurons in the MVN and SVN were located about 1–2 mm caudal and 0–2 mm lateral from the center of the abducens nucleus (3, 29, 32). Special care was taken to ensure that the same neuron was recorded during the experiment, similar to our previous studies (28, 29, 32, 45). That is, we compared the shape of the recorded action potentials during the entire experiment. Canal convergent inputs or tests of other characteristics that were specific for the particular neuron were repeated at different times and compared during off-line analyses. Firing rates of each neuron with the animal in the upright position for 20 s at the onset of each test were recorded to monitor the stability of the resting firing rates. The resting firing rate was computed and plotted as a function of time off-line. We assumed that the unit recording was stable during the experiment if the SD about the mean value did not dramatically change (Figure 2). The units had differing spontaneous firing rates (FR), which in some cases were gradually modified during the experiments (Figures 2A,B). Unit #11 (Figure 2A) had only minor variations in the average firing rate, but the firing rate variations substantially increased after 7.5 h of recording. Unit #52 (Figure 2B, circles) had some variation of the average firing rate, which dramatically increased from 28.9 ± 3.3 to 38.5 ± 4.5 imp/s (*p* < 0.01) after ≈7.5 h of testing. Changes in firing rate of both neurons indicated that data obtained later than 7.5 h from the onset of the experiment could not be used for analyses.

### Experimental Protocol

Vestibular-only and VPS neurons were identified during oscillation of the monkeys by hand about a spatial vertical axis. An audio monitor and an oscilloscope were used to select neurons whose firing rates were associated only with vestibularly induced eye and head velocity and had no obvious relation to eye position or ocular pursuit (3, 29, 46, 47). Thus, modulation of the firing rate of VO and VPS neurons was about the same regardless of whether the VOR was tested in darkness, in light, or suppressed or canceled by oscillation of the animal in phase with the visual surround. To determine that there was no relation to eye position, visually driven eye velocity, ocular pursuit, or spontaneous eye movements, the neuron was further examined off-line by color coding the firing rate on an *x*–*y* plot to determine its eye position and eye velocity sensitivity. This method was utilized to exclude all neurons whose eye position sensitivity was >0.05 imp/deg (32).

To identify whether the neurons were VPS cells, unit activity was synchronized with the beginning of 10–50 saccades of approximately identical amplitudes (25° ± 3°) in all directions. Unit firing rate during saccades was analyzed using raster diagrams and superimposed average algorithms (Figures 2C,D). The pauses in activity were approximately overlaid for each saccade and the onset of the pause slightly preceded saccade onset and ended when the saccade reached peak velocity. Some units had pauses only for saccades to the left or right, but not during vertical saccades. Other units had pauses associated with saccades in all directions. This test was essential because neurons that have pauses during saccades only in a particular direction or during a portion of the saccadic interval frequently could not be detected on-line as VPS neurons using video or audio monitors (Figures 2C,D).

### Tests to Determine Neuronal Sensitivities

Identification of canal and otolith-related inputs for all neurons were reported elsewhere (32). Each unit was also tested for convergent inputs from muscle proprioceptors by pressing on the muscles in the neck, arms, legs, and body and listening for changes in firing rate on an audio monitor or by noting changes in firing frequency on an oscilloscope. All units that had detectable convergent proprioceptive inputs were not studied further. This technique does not guarantee that all units with neck proprioceptive inputs were excluded from the analysis (48), but it eliminated a significant number of such neck-related neurons (32, 49). Therefore, the VO and VPS neurons tested in this study may represent a specific sub-group of units tested by others (50–52).

It was previously demonstrated that VO and VPS neurons have approximately the same sensitivity to sinusoidal oscillation at 0.2 Hz and above in darkness, in light and during suppression of the VOR gain (46, 53). It was also demonstrated that visual following of an OKN stimulus at frequencies 0.05 Hz only are coded in such neurons (54). These studies concentrated only on one aspect of velocity storage and it is not clear whether only VO neurons were considered. To clarify, neurons in this study were tested during sinusoidal oscillation at 0.2 Hz in darkness, in light (OKN drum stationary in space while animal is oscillated in yaw), and during suppression of the VOR (OKN drum was linked to yaw axis and, therefore, animal and drum were oscillated in yaw in phase). Neurons were also tested by sinusoidal oscillation of OKN drum in yaw at frequencies from 0.01 to 0.5 Hz. When it was possible, rotational VOR tests were also performed at various frequencies.

To determine symmetry of neuronal responses for rotations in alternate directions the animals were rotated at 60°/s for 5 s and then stopped in light for 5 s to eliminate post-rotatory nystagmus and to determine the neuronal sensitivity to step rotation in ipsilateral and contralateral directions. The light was extinguished and after 2 s rotation was repeated in the alternate direction. Average neuronal firing rates over the first 2 s were computed from 10 rotations in each direction.

### Tests to Determine the Relation of Neuronal Firing Rate to Velocity Storage in One Dimension

The animals were seated in a rotating chair with the head fixed in the stereotaxic position. The lights were extinguished and the animals were rotated about a yaw axis at a constant velocity 60°/s. The slow phase eye velocity was close to that of head velocity at the onset of rotation but gradually decayed to 0 over time. The rotation was stopped to induce post-rotatory nystagmus. The pre- and post-rotatory profiles were similar but opposite in polarity. After a brief 7-s period in light, similar rotation in the opposite direction induced oppositely directed per- and post-rotatory nystagmus. After a 10-min rest in light, the units were tested over a range of velocities from 30 to 180°/s in 30°/s increments, starting at 60°/s.

The monkeys were also rotated side-down about the spatial vertical axis to induce vertical (pitch) nystagmus, as described above. Additionally, some units were tested by tilting the animal right side down and then rotating it in the yaw 45° in CW and CCW directions to bring the left anterior–right posterior or right anterior–left posterior canal pair, respectively, to the plane of rotation. A short period in light for 4–5 s during post-rotatory nystagmus was used in some experiments (6, 8) to determine the neural activity during rapid time constant variations.

### Tests to Determine the Relation of Neuronal Firing Rate to Velocity Storage in Three Dimensions

Optokinetic nystagmus was induced by rotation of the optokinetic drum about the animal’s yaw axis with the axis of rotation coincident with the spatial vertical axis. The OKN drum was rotated at a constant velocity for 30 s and then OKAN was recorded in darkness while the animal was in the upright position. After a brief (5 s) exposure to light, OKN, and OKAN were recorded in the opposite direction. Typically, OKN was induced at 60°/s, although some neurons were tested at velocities of 30, 90, and 120°/s. To test how neurons code spatial orientation of the velocity storage, OKN and OKAN were recorded with the animal tilted side down or prone-supine in 30° increments up to 90°. In these positions OKAN induced by rotation of the visual surround about the animal’s yaw axis was initially only about the animals head-yaw, but then cross-coupled to pitch or roll nystagmus, depending on the direction of the animal’s tilt with regard to gravity.

It was previously shown that eye velocity declines along an eigenvector of velocity storage, which tends to shift toward the spatial vertical (12). Thus, correlation of the time constant of the unit firing rate with the time constant of the primary yaw eye velocity, which is dependent on tilt angle, indicated that the neuron was a VO or VPS neuron that coded only the yaw component of velocity storage. Then correlating the firing rate with the cross-coupled components would indicate that the unit coded the spatial properties of the velocity storage integrator.

### Data Processing and Analyses

Eye positions were digitally differentiated to determine slow phase eye velocity after saccades had been identified (55), and marked off from further analyses. The accuracy of desaccading was visually verified, and in some cases (<5%) manually corrected.

The neuronal data were converted into an instantaneous frequency which is an inverse of the inter-spike interval (imp/s) to measure the dominant time constant of the firing rate during vestibular and OKN. The neuronal data were expressed as a histogram with a moving average. That is, the neuronal firing rate was binned in 25 ms intervals. Then values, of the first five intervals were averaged and assigned to the middle (third) intervals. The first interval was then omitted and one more interval added to the average and the averaging procedure was repeated (3). Both methods of neuronal data presentation had similar values of average firing rate and sensitivity in response to sinusoidal oscillations at frequencies below 0.5 Hz, but substantially reduced data variation due to the moving average algorithm.

Vestibular per- and post-rotatory nystagmus induced in the upright were treated as dual time constant processes, where the first time constant of the cupula is known (4 s) and second time constant of the velocity storage integrator was determined. These algorithms of data fit were previously described (3, 6, 12). The decaying slow phase eye velocities and neuronal firing rate from the onset of OKAN till the time when they reached steady state level were fitted with a single time constant algorithm to measure the velocity storage of the OKAN induced in the upright position. OKAN induced when the animal was tilted side-down, forward, or backward were treated as dual time constant processes: the first, the time constant of the integrator and the second, the time constant of the cross-coupled component of eye velocity. The data were fit assuming that both time constants were unknown.

### Statistical Analyses of the Data

The significance of the sinusoidal fit through the data was tested with an F-statistic, which is a reduced case of the general analysis of variance (ANOVA) (42, 56). A standard two-tail *t*-test was used to compare two groups of data. An ANOVA was used to compare more than two groups of data. If the general ANOVA showed significant differences, between data sets, then each between-group degree of freedom was analyzed separately by developing orthogonal contrasts. In this case, the results of the test were adjusted with a Scheffe approach (57).

## Results

### Response of VO and VPS Neurons to Slow Phase Eye Velocity

The firing rates of 33 VO and 8 VPS neurons were studied during nystagmus induced by rotation at a constant velocity in darkness (38 units) or during OKAN (32 units) to determine their relationship to velocity storage. Fourteen additional neurons modulated their activity in relation to velocity storage but were only partially tested. Their neuronal activity was lost before a sufficient number of tests could be performed to determine their time constants as a function of head orientation or rotational velocity. Thus, after a neuron was selected for recording, there was a 75% chance that the neuron would survive over the entire testing period. Data from these 14 neurons were used in this study only to determine their sensitivity to step rotation.

Forty-one VO and VPS neurons formed the body of data that were extensively studied. These neurons were subjected to all tests to determine their relationship to velocity storage. One anterior canal-related neuron had no significant modulation. One posterior canal-related VPS cell was modulated significantly with each rotation, but its time constant was ≈4 s and was independent of the eye velocity response, which had the time constant of velocity storage (15–25 s). This suggests that a small population of VPS neurons is related to the direct vestibular pathway in support of the model structure (Figure 1, G_1_) and could provide input to the integrator (Figure 1, G_0_).

### Neuronal Firing Rates during VOR in Dark, in Light, and during Cancelation of the VOR with Sinusoidal OKN

The 22 VO and VPS neurons were recorded during steps of rotation in darkness (VOR dark), in light (VOR light), in a subject stationary visual surround (VOR cancelation), and during sinusoidal optokinetic stimulation (OKN). The average VOR gain was unity when tested in darkness or in light. The VOR gain decreased to 0.31 ± 0.25 during the cancelation paradigm (*p* < 0.05, ANOVA). The gain of the ocular following during sinusoidal OKN was 0.60 ± 0.15, similar to the gains reported earlier (6, 8) and is consistent with the model (Figure 1).

**Figure 3:**
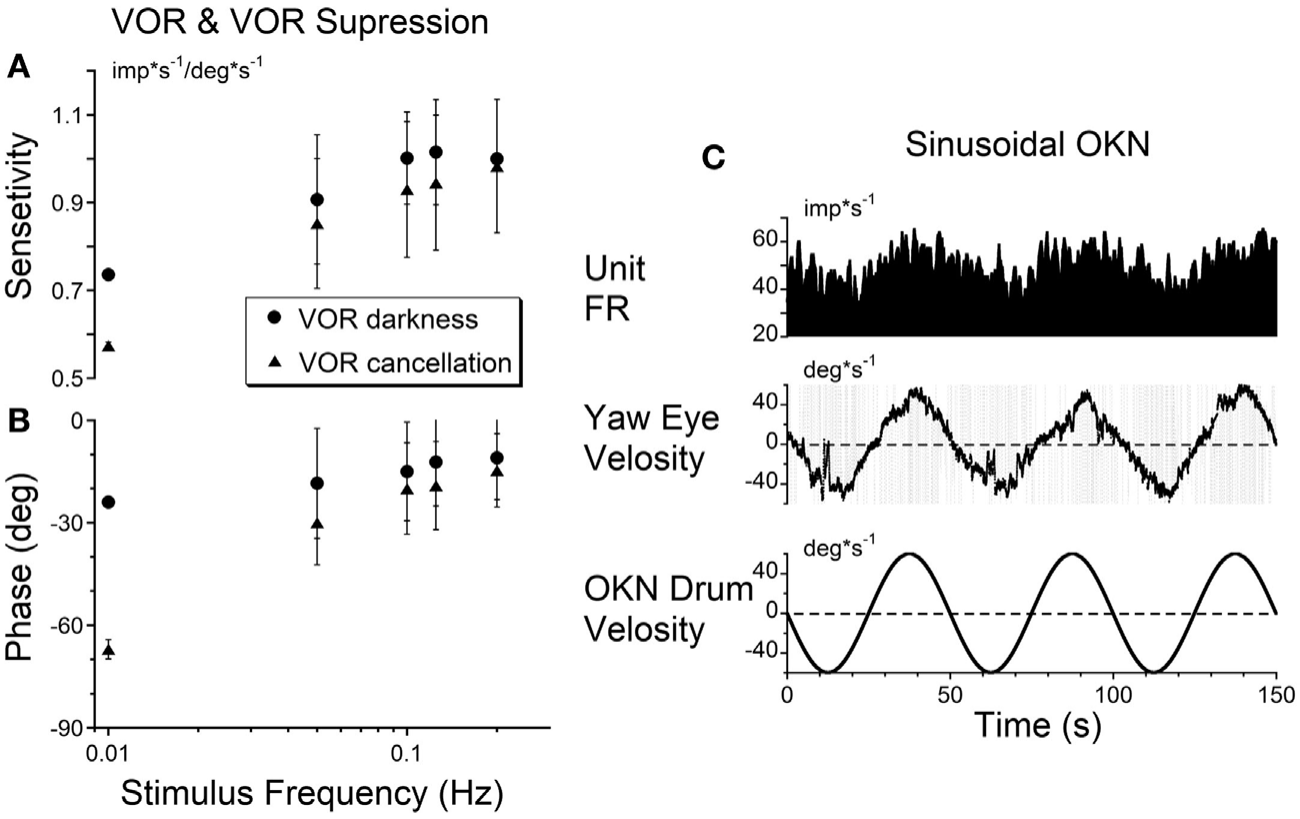
**(A,B)** Gains **(A)** and phases **(B)** obtained from a typical vestibular-only neuron during sinusoidal oscillation in darkness (VOR darkness, filled circles) and oscillation in light with the subject-fixed visual surround (VOR cancelation, filled triangles). **(C)** Activity of the same neuron recorded during sinusoidal oscillation of optokinetic surround at 0.02 Hz [optokinetic nystagmus (OKN)].

The sensitivities of the firing rate of the VOR cancelation was not significantly different from that of VOR in dark (Figures 3A,B) or in light (not shown) for oscillation at 0.1 Hz and above (*p* = 0.561), but became progressively larger at 0.01 Hz, the frequency where velocity storage would play a significant role for rotation in darkness (Figures 3A,B).

Twenty of the 21 tested units did not respond to oscillation of the visual surround during OKN at 0.2 Hz. This reflected their insensitivity to cortically induced visual activity. However, the unit activity was modulated at lower frequencies of optokinetic stimulation (Figure 3C), indicating that the velocity storage integrator was sensitive to optokinetic stimulation at low frequencies (6). That is, the vestibular neurons were likely responding to activation through the subcortical pathway through the nucleus of the optic tract (NOT) (58, 59), not through the brainstem pathways to the oculomotor system through the flocculus. Regardless, Figure 3C supports the idea that VO and VPS neurons code velocity storage.

One neuron, however, had a small but significant modulation of the firing rate during OKN stimulation at 0.2 Hz (0.101 imp*s^−1^/deg*s^−1^). Since the firing rate of this neuron was not related to eye position and velocity, this unit was identified as a VO neuron. Thus, a few VO neurons may have some access to the direct optokinetic input to velocity storage (Figure 1A, G_2_).

### Sensitivity to Angular Rotation in the CW and CCW Directions

**Figure 4:**
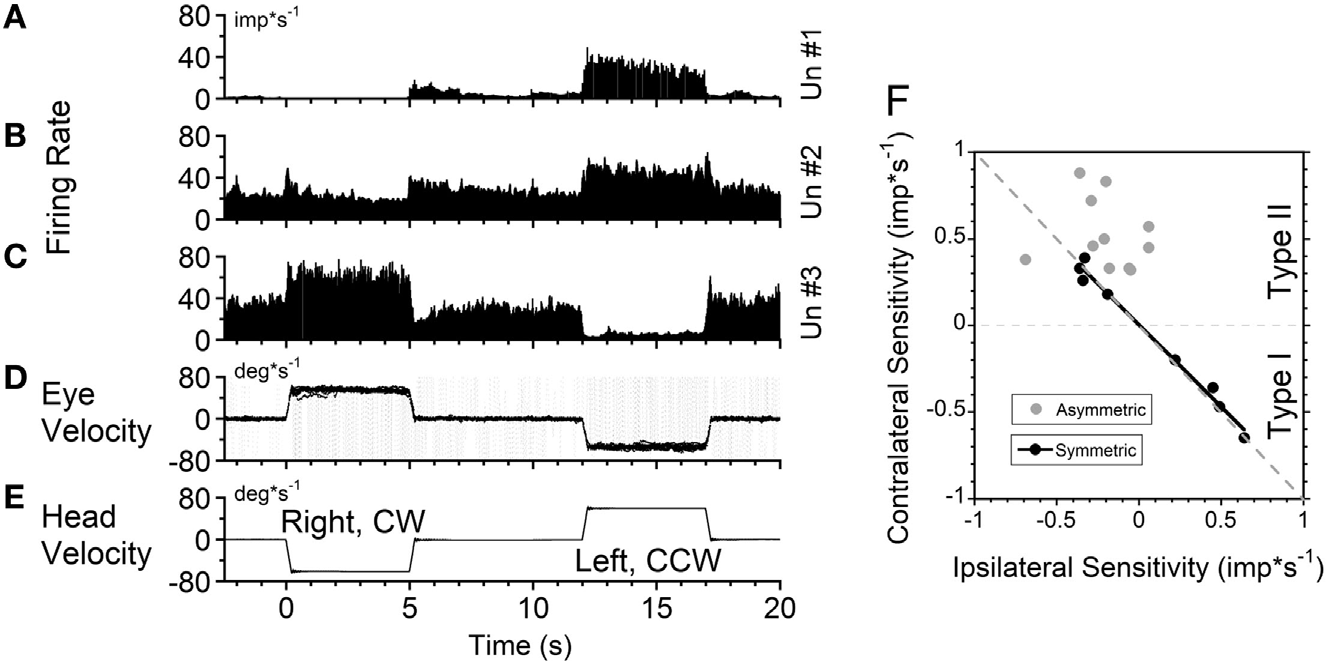
Changes in neuronal firing rate of three vestibular-only neurons located in the left **(A)** and right **(B,C)** hemispheres in response to head rotation to the right [**(E)**, negative velocity] and to the left (positive velocity) in darkness. **(D)** Superimposed eye velocities for 10 repeated rotations. Gray—original eye velocities. Black—slow phase eye velocities. See details in the text. **(F)** Relationship between average sensitivity to rotation toward ipsilateral (abscissa) and contralateral (ordinate) sides. Black symbols—neurons with identical sensitivities to rotation in both directions (*p* < 0.05). Gray symbols—neurons with asymmetrical sensitivities.

Seventeen VO and six VPS neurons were sensitive to head rotation with the step of velocity in yaw (Figures 4A–C). One neuron in the left vestibular nuclei had a high sensitivity for rotation to the ipsilateral side (left, CCW, Type I, Figures 4D,E). Its firing rate ceased during rotation toward the contralateral side (right, CW) (Figure 4A). For such neurons, only the ipsilateral sensitivity could be computed (Table 1, dashes in values). There were three Type I and one Type II VO neurons that were sensitive to rotation only in one direction (17%, 4/23).

**Table 1:** Neuronal sensitivity obtained by rotation with steps of velocity of 60°/s.

Monkey #	Unit #	Unit type	Sensitivity to rotation		*p*-Value	Canal input
			Ipsilateral	Contralateral		
M1	18	Vestibular-only (VO) 1	0.79 ± 0.26	–	<0.0001	LCi
M3	11	VO 1	1.01 ± 0.03	–	<0.0001	LCi and ACi
M3	16	VO 1	0.94 ± 0.26	–	<0.0001	LCi
M1	3	VO 2	0.06 ± 0.08	0.45 ± 0.08	<0.0001	ACi
M1	61	VO 2	–	0.76 ± 0.10	0.011	ACi
M1	64	VO 2	−0.06 ± 0.14	0.33 ± 0.11	<0.001	PCi
M1	66	VO 2	−0.21 ± 0.07	0.50 ± 0.13	<0.0001	ACi,c
M2	4	VO 2	−0.20 ± 0.09	0.83 ± 0.18	<0.0001	LCc and RAi
M3	12	VO 2	−0.28 ± 0.05	0.46 ± 0.14	0.003	LCc
M3	22	VO 2	−0.36 ± 0.12	0.88 ± 0.10	<0.0001	LCc
M3	29	VO 2	−0.05 ± 0.10	0.32 ± 0.10	<0.0001	PCc
M3	33	VO 2	0.06 ± 0.11	0.57 ± 0.16	<0.0001	ACi and PCi
M3	53	VO 2	−0.29 ± 0.07	0.72 ± 0.13	<0.0001	LCc and ACc
M3	23	VPS2	−0.18 ± 0.09	0.33 ± 0.06	<0.0001	PCi
M3	43	VPS2	−0.69 ± 0.41	0.38 ± 0.34	0.017	ACi
M1	58	VO 1	0.45 ± 0.12	−0.36 ± 0.15	0.218	LCi
M3	30	VO 1	0.49 ± 0.09	−0.47 ± 0.08	0.622	LCi and PCi
M3	45	VO 1	0.64 ± 0.12	−0.65 ± 0.11	0.948	LCi and ACi
M3	41	VPS1	0.22 ± 0.06	−0.20 ± 0.06	0.461	LCi
M1	67	VO 2	−0.33 ± 0.09	0.39 ± 0.14	0.285	PCi
M1	62	VPS2	−0.36 ± 0.25	0.33 ± 0.09	0.690	LCc
M3	58	VPS2	−0.34 ± 0.14	0.26 ± 0.09	0.174	ACi
M3	59	VPS2	−0.19 ± 0.07	0.18 ± 0.04	0.934	PCi

*Canal inputs were determined in our previous study (32). LCi, ACi, and PCi are lateral, anterior, and posterior canals inputs from the ipsilateral side. LCc, Acc, and PCc, inputs the same canals from contralateral side. Ten rotations were performed in each direction*.

Another neuron, located in the right vestibular nuclei, had a comparable response to contralateral rotation (left, CCW, Type II), but a much smaller response to rotation toward the ipsilateral side (Figure 4B). The sensitivities of such neurons are shown in Table 1 (second block from the top) and are summarized in Figure 4F (gray symbols). All these neurons (48%, 11/23) were Type II neurons (9 VO and 2 VPS), and for all except one (Un #43; Table 1) the sensitivity to contralateral rotation was larger than the sensitivity to the ipsilateral side.

Thus, 65% of VO and VPS neurons in our study had an asymmetric response to rotation. There was no difference between VO and VPS neurons or whether the units received lateral canal, vertical canal, or an otolith input. Among 10 recorded neurons, seven received only vertical canal-related input (Table 1 (32)). Three other neurons received lateral canal-related input, two of which also had convergent vertical canal-related inputs. Thus, asymmetry in sensitivity did not correlate with whether neurons were VO or VPS cells or whether they received lateral canal, vertical canal, or otolith inputs. A third neuron, located in the right vestibular nuclei, had comparable increases in firing rate during ipsilateral (CW, Type I) and decreases in firing rate during contralateral rotations (Figure 4C). The sensitivity of nine such neurons is shown on the bottom portion of Table 1.

Figure 4F summarizes responses of neurons with symmetrical (black circles) and asymmetrical (gray circles) sensitivities to rotations in both directions. By definition, neurons with symmetrical responses should lay along a diagonal line that goes from the unity sensitivity for rotation toward the ipsilateral and contralateral sides (gray dashed line). Neurons with asymmetrical sensitivity clustered in the upper left quadrant indicating that they all were Type 2. Four neurons with no sensitivity to rotation in one direction are not shown but they would lay along the ordinate 0 (dashed black horizontal line). Thus, we did not find any Type I neurons which responded to rotation in both directions asymmetrically.

Among nine neurons with symmetrical responses, four were Type I and five were Type II (Table 1, bottom two blocks). The relationship of the firing rate to the ipsilateral and contralateral rotations of these neurons is summarized in Figure 4F (black symbols). As expected, they are very close to the gray dashed symmetry line, i.e., their responses were approximately equal for CW and CCW rotations.

### The Relation of the Neuronal Discharge to the Time Constant of the Yaw Angular Vestibulo–Ocular Reflex (VOR) Tested with Angular Rotations and OKN of Constant Velocities

**Figure 5:**
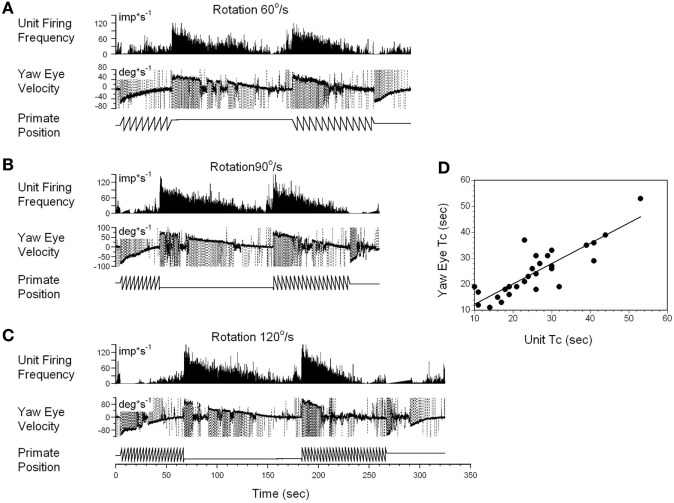
Changes in the firing rate of a typical vestibular-only (VO) neuron that codes the yaw component of velocity storage during head rotation at 60°/s **(A)**, 90°/s **(B)**, and 120°/s **(C)**. **(A–C)** In each section, after being in darkness for 5 s, the animal was rotated to the left (counterclockwise) and then stopped while in darkness (primate position, tooth-like, and constant levels, respectively). Then rotation was repeated in the opposite (clockwise) direction. This sequence induces per-rotatory nystagmus to the right (Negative Eye Velocity) which gradually declined to 0. When rotation was stopped, it induces post-rotatory nystagmus in the opposite direction (positive eye velocity). This sequence of per- and post-rotatory nystagmus was repeated in the opposite directions. The experiment animal was extremely drowsy, and there was a sudden decline of slow phase eye velocities to 0. Drowsiness, however, did not the affect firing rate of this VO neuron. **(D)** Relationship between the time constants of velocity storage determined from slow the phase eye velocity (ordinate) and from the neuronal firing rate (abscissa). Each data point on this graph represents a time constant measured for the oculomotor response and corresponding neuronal response. This is shown in the two responses in graphs **(A–C)**. The black line is the linear regression of the data.

A typical example of the changes in the neuronal firing rate of a VO neuron induced by head rotation at 60, 90, and 120°/s in darkness is shown in Figures 5A–C. During this test, the animal was extremely drowsy and slow phase eye velocity was frequently suppressed to 0. The unit firing rate decrease, however, was not affected by this, and the gradual decrease to the bias level was the same as when the animal was alert (not shown). Periods of drowsiness were removed and time constant of slow phase eye velocity was compared to that of changes in neuronal firing rate (Figure 5D). Thus, the changes in the time constant of neuronal firing closely approximated the VOR time constant, which was the time constant of velocity storage. Additionally, drowsiness periods were occasionally observed during recording of almost every neuron, but drowsiness never affected the firing rate of the VO neurons.

Not every VO and VPS neuron had its firing rate related to velocity storage. Some neurons that had asymmetrical responses to CW and CCW rotations fell into this group, at least for the direction in which the sensitivity was lower (not shown). This supports the idea that the time constant of velocity storage for rotations in alternate directions is controlled by different neurons. This could also be the reason why the time constant for rotations in alternate directions are always slightly different, regardless of whether rotation was to the left or to the right.

**Figure 6:**
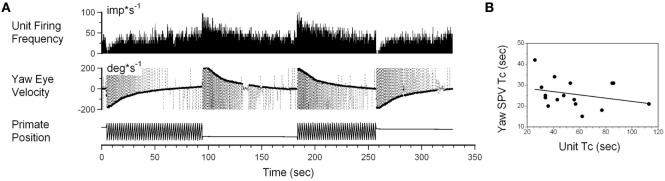
**(A)** Changes in the neuronal firing rate induced by head rotation about a spatial vertical axis of vestibular-only neuron that does not code the yaw component of velocity storage. See also legend to Figure 5 for details. **(B)** Changes in the time constant determined from yaw slow phase eye velocities (ordinate) did not correlate with changes determined from the neuronal firing rate (abscissa).

Interestingly, while the firing rate of some neurons was altered by angular rotation, the time constant of the neuronal response was not correlated with the time constant of velocity storage as determined by the slow phase eye velocity. An example is shown in Figure 6A. This neuron increased its firing with leftward slow phase eye velocity (Figure 6A, yaw eye velocity upward) and decreased firing with rightward eye velocities (negative values). Once again small drowsiness intervals during the post-rotatory response to the left did not affect the neuronal firing rate. The time constants obtained from neuronal and oculomotor responses, however, were not correlated with any specific direction of head rotation (Figure 6B, *p* > 0.05). This neuron was significantly modulated during rotation at constant velocities, and the changes in its firing rate were not associated with the yaw component of velocity storage.

**Figure 7:**
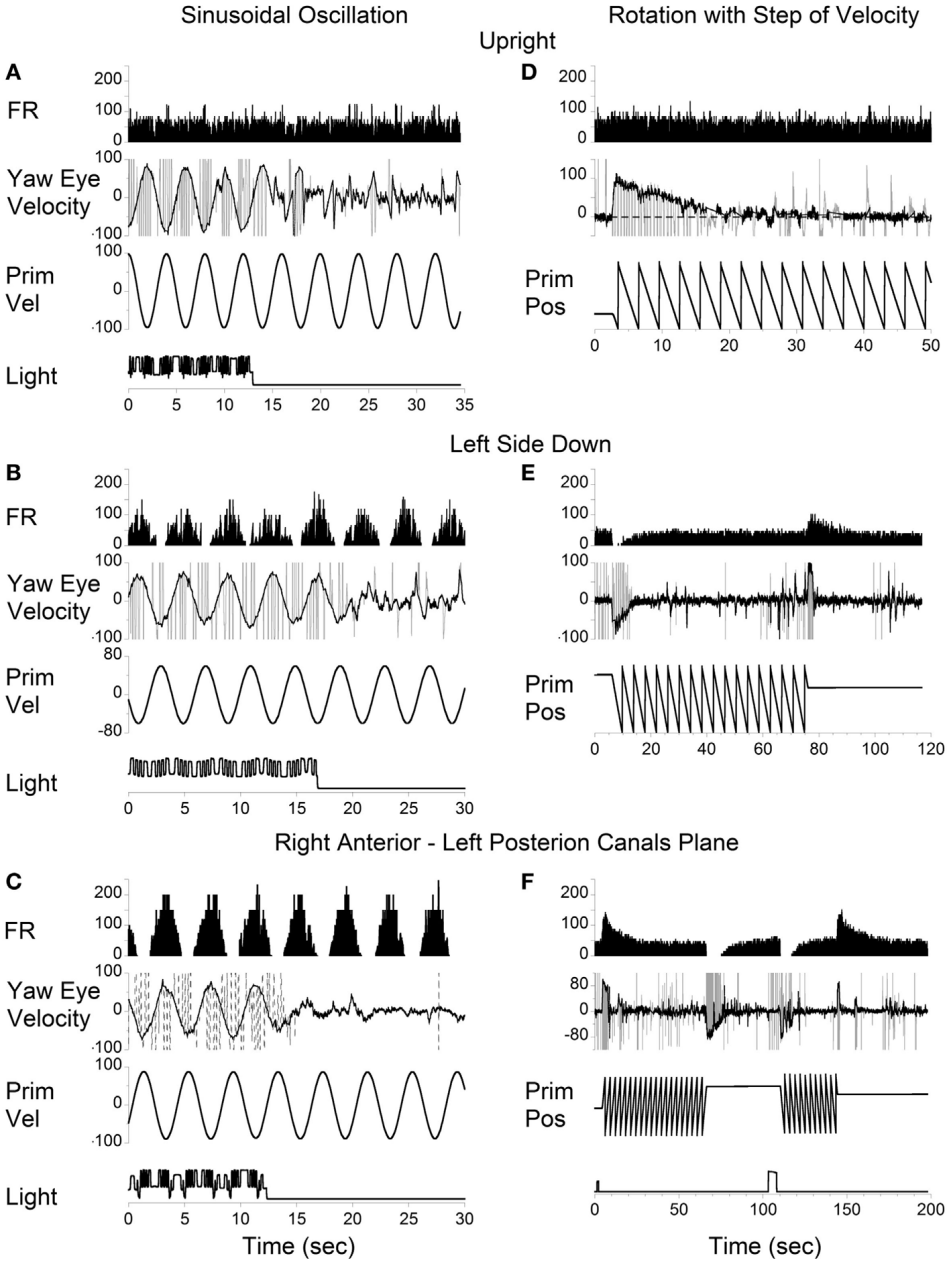
Left posterior canal-related neuron tested by sinusoidal oscillation **(A–C)** and by step rotation **(D–F)** about a spatial vertical axis while the animal was upright **(A,D)**, tilted left side down **(B,E)**, or tilted left side down and then rotated 90° counterclockwise in yaw to bring the right anterior–left posterior canals to the plane of rotation **(C,F)**.

Vertical canal-related neurons typically did not respond to sinusoidal oscillation about the spatial vertical while upright (Figure 7A). Their modulation became significant when the animal was oscillated about the spatial vertical axis after being tilted left side down (Figures 7B,E). Furthermore, the modulation was even larger when the oscillation was in the plane of the left posterior canal that activated this neuron (Figures 7C,F). This demonstrates that the firing rates of some neurons are specific to the canal plane that innervates these neurons. It suggests that the activity of the velocity storage in these neurons is actually coded in the canal rather than in the head coordinates. Since oculomotor responses are coded in the head coordinates (see Introduction), signals coding for the firing rates of this type of neuron should be converted from the canal into head coordinates.

**Figure 8:**
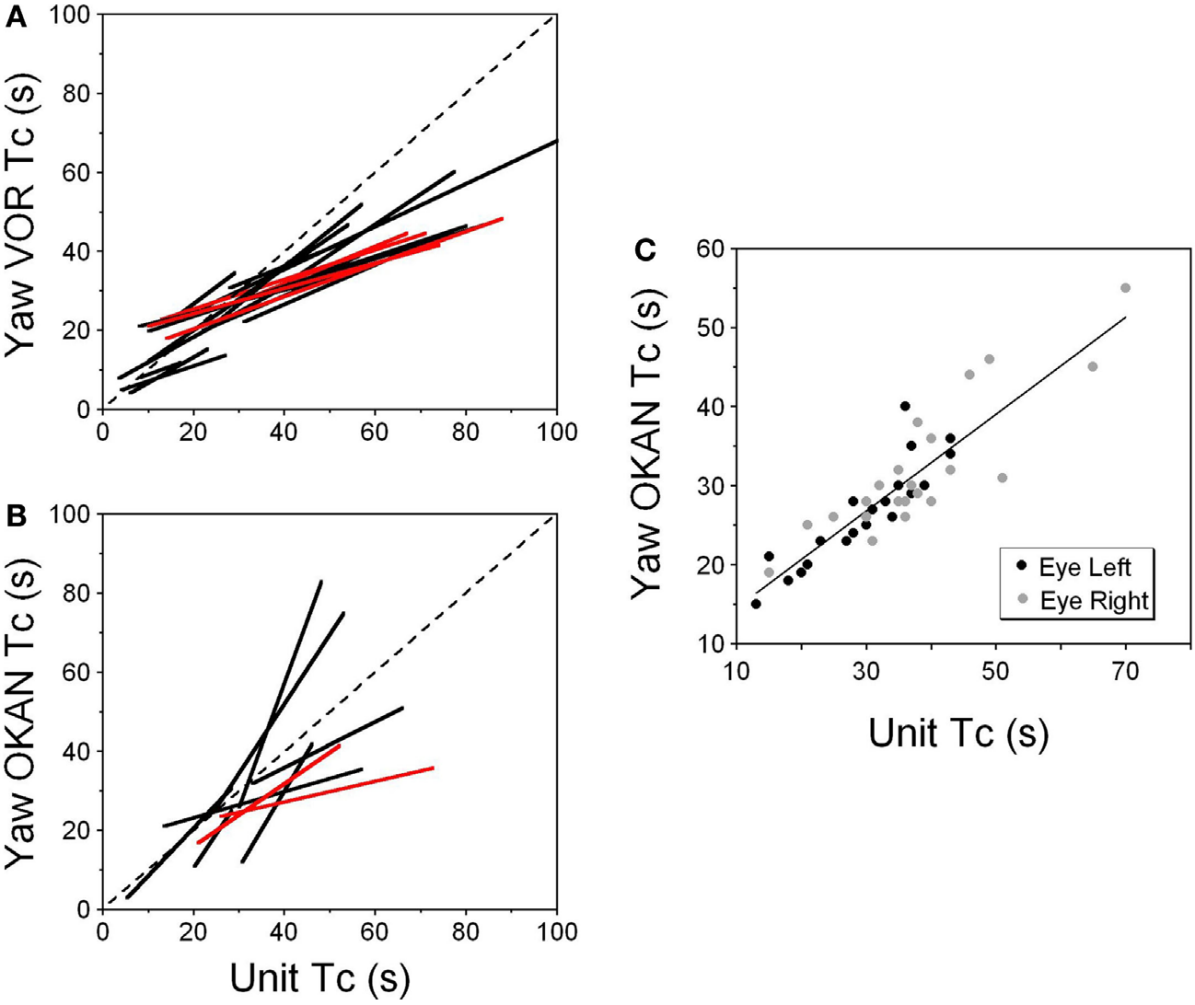
**(A,B)** Relationship of the time constant of velocity storage measured from the yaw slow phase eye velocity (abscissa) during vestibular nystagmus **(A)** and optokinetic after-nystagmus (OKAN) **(B)** and measured from the changes in the neuronal firing rate (ordinate). The individual lines are regression lines for the vestibular-only (black) and VPS (red) neurons. **(C)** Relationship between the time constant of the yaw component of velocity storage tested with 60°/s optokinetic nystagmus/OKAN while the head was tilted side down at various degrees up to 90°. The time constant of the decline in neuronal firing is shown in Figure 9.

Correlation of the time constants determined from neuronal and yaw oculomotor responses was significant for 15 VO neurons. Figure 8 demonstrates the linear regression lines determined for these units (Figure 8A, black lines). The beginning and the end of each line indicates the range within which each neuron was tested. On average, the regression lines from VO neurons were clustered below the dashed line which indicates the 1:1 ratio of these parameters. An average slope was 0.593 ± 0.213 indicating that the time constant of velocity storage was longer in the activity of the VO neurons than in the oculomotor responses. There was only one VPS neuron, whose time constant was comparable to the accepted cupula time constant (≈4.5 s) and was independent of the time constant of VOR.

Drowsiness affectively changed the firing patterns of the VPS cells (not shown) (3, 60). Thus, the firing rates of VPS neurons are associated with oculomotor behavior, rather than with manifestations of velocity storage. Regardless, the activity of the five VPS neurons was significantly modulated by head rotation about the animal’s yaw axis. When the time constant of the velocity storage obtained at different rotation velocities was plotted against the time constant of the neuronal firing rate, the two parameters were correlated (Figure 8, red lines). An average slope of the regression line for VPS was 0.307 ± 0.059 which is smaller than that for VO neurons (*p* = 0.0092).

Thirty two neurons were tested for the relationship of their firing rate to the time constant of OKAN. Among them were six vertical canal-related and one lateral canal-related neuron that did not respond to OKAN induced by drum rotation about the animal’s yaw axis in the upright. Twelve neurons did respond to OKAN but causal relationship was not certain. Furthermore, there was not enough data to determine the relationship between the time constants of neuronal and oculomotor responses. The remaining 13 neurons were also related to yaw OKAN and were extensively tested. There was a significant correlation between the decaying time constant of the changes in the neuronal firing rate and the slow phases of yaw nystagmus for 11 VO (Figure 8B, black lines) and the two VPS neurons (Figure 8B, red lines). This was similar to the previously described relationship to rotatory nystagmus, some neurons that had a clear correlation of their neuronal firing rate to the yaw component of OKAN did not receive convergent inputs from the lateral but only from the vertical canals.

The neurons whose firing rates were related to the yaw component of OKAN were also tested by sinusoidal oscillation of the visual surround at low frequencies. All of the tested neurons were modulated by visual surround oscillation at 0.02 Hz (Figure 3C), and about half of them were also modulated at 0.05 Hz oscillation. This was an additional confirmation that the firing rate of these neurons was related to velocity storage.

### Relation of the Neuronal Discharge to the Orientation Properties of Velocity Storage

Thirteen neurons were tested by inducing yaw OKN/OKAN when animals were upright or tilted sideways up to 90° (Figure 8B). Ten neurons were classified as VO neurons and three were classified as VPS neurons. Twelve of the 13 (12/13) neurons had their firing rate related to the horizontal component of the OKN/OKAN. Three neurons received convergent inputs only from the lateral canal on the ipsi- (1, Type I) or contralateral (2, Type II) side. Three neurons received convergent inputs from lateral (2 ipsi-, 1 contralateral) and anterior (2 ipsi-, 1 contralateral) canals. Six neurons did not receive any convergent inputs from the lateral canals, only from vertical canals (2 ipsi- anterior, 1 ipsi- posterior and 3 contralateral posterior canals), and yet they firing rates were related to yaw rater then pitch OKN/OKAN.

**Figure 9:**
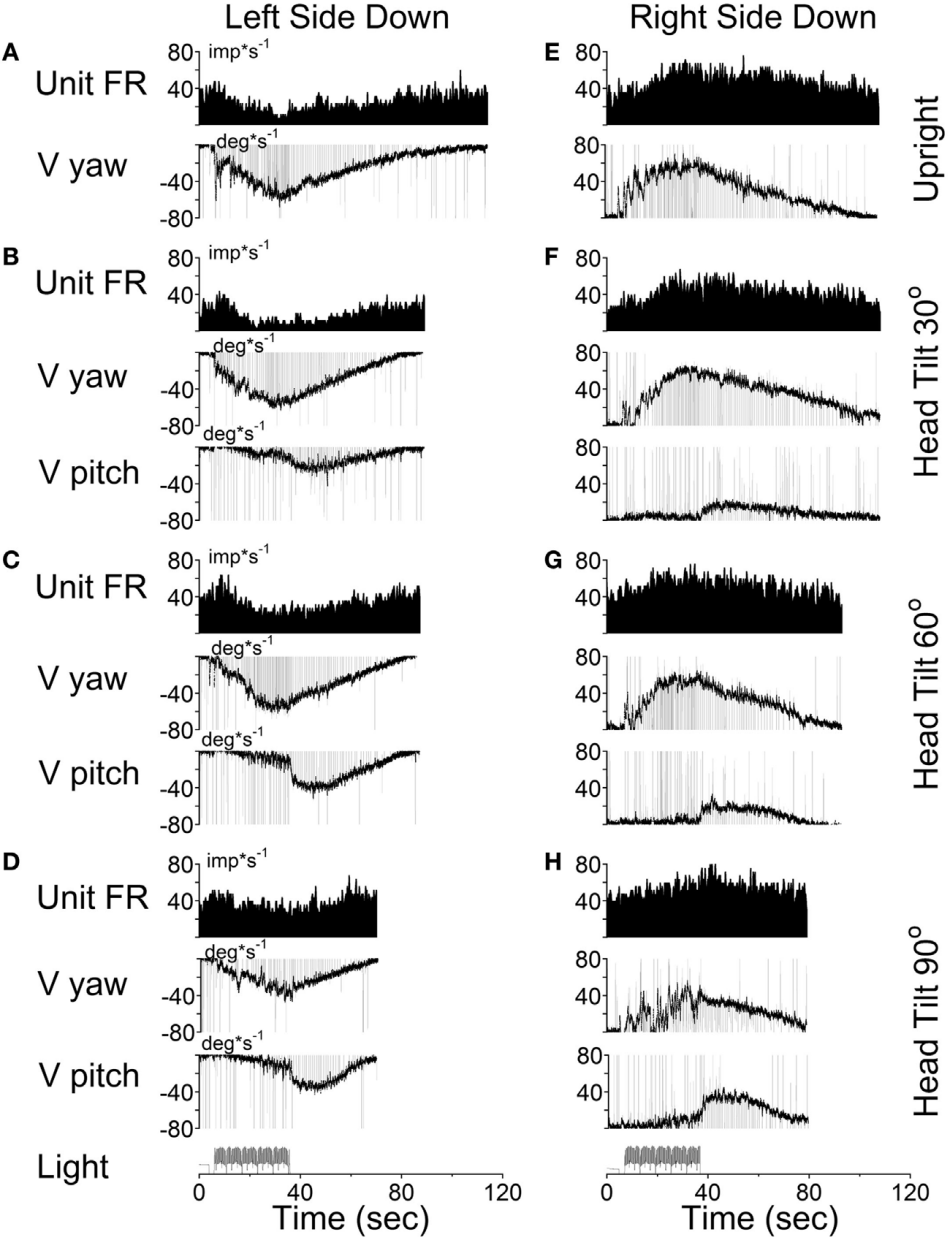
Changes in the firing rate (Unit FR) of a Type II vestibular-only neuron that receives convergent inputs from the otolith and the contralateral, lateral, and anterior canals. The neuron was tested in the left side down **(A–D)** and right side down **(E–H)** positions. For each position, the neuron was tested while upright **(A,E)** or while tilted 30° **(B,F)**, 60° **(C,G)**, or 90° **(D,H)**. The firing rate decrease in response to optokinetic nystagmus (OKN)/OKAN to the right [**(A-D)**, unit FR] and increased in response to OKN/OKAN to the left [**(E–H)**, unit FR]. In a tilted position, the duration of the yaw component of OKAN decreased with the tilt angle (V yaw), and there was a cross-coupled vertical component (V pitch). Changes in the firing rate of this neuron were only associated with changes in V yaw.

**Figure 10:**
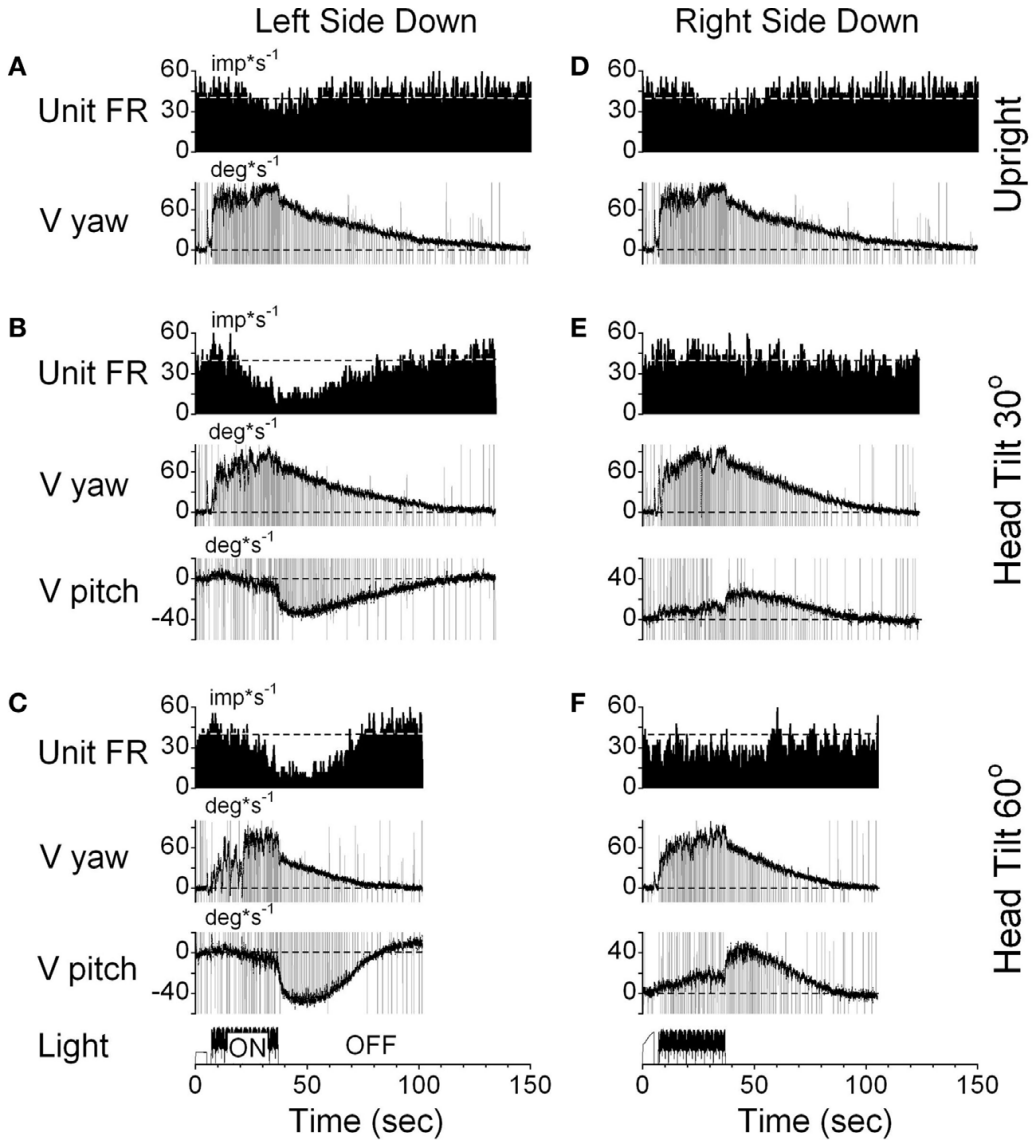
Changes in the firing rate (Unit FR) of a VPS neuron that received convergent input from the contralateral posterior canal during optokinetic nystagmus (OKN)/OKAN in the left side down **(A–C)** or right side down **(D–F)** positions. For each position, the neuron was tested while upright **(A,D)** or while tilted 30° **(B,E)** or 60° **(C,F)**. **(A,D)** are replicated from the same data. V yaw is the yaw slow phase eye velocity and V pitch is the slow phase pitch eye velocity. ON denotes the OKN interval, and OFF denotes the OKAN interval. Data in panel **(D)** are duplicate of panel **(A)**. This was done to organize data in columns to simplify comparison.

**Figure 11:**
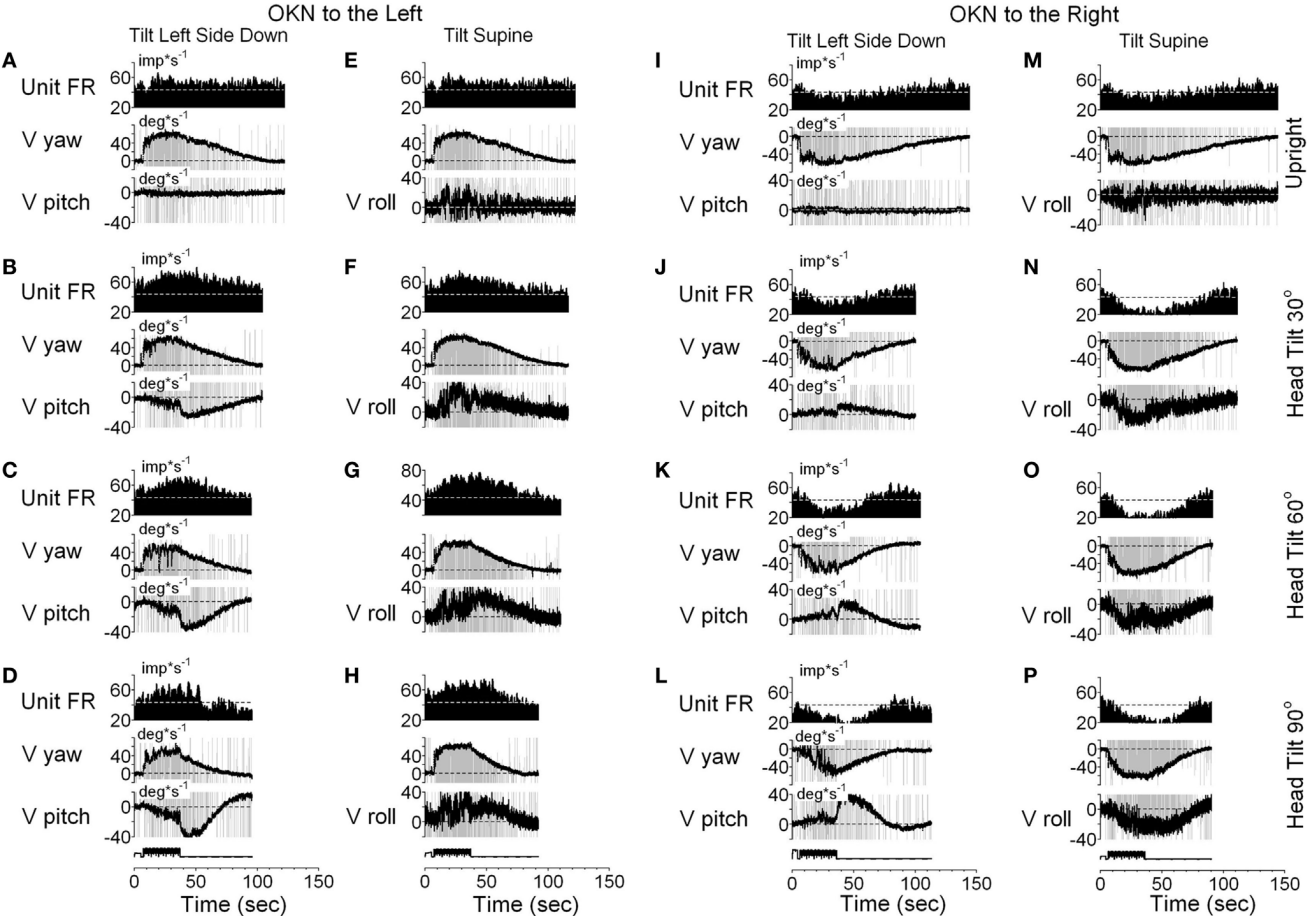
Unit firing rates and yaw (V yaw), pitch (V pitch), and roll (V pitch) slow phase eye velocities for a VPS neuron that received input from the ipsilateral anterior canal during optokinetic nystagmus (OKN)/optokinetic after-nystagmus to the left **(A–H)** and to the right **(I–P)**. This neuron was tested in the left side down **(A–D,I–L)** and supine **(E–H,M–P)** positions. In each position, the neuron was tested while upright **(A,E,I,M)** or while tilted 30° **(B,F,J,N)**, 60° **(C,G,K,O)**, or 90° **(D,H,L,P)**. Data in panel **(E)** are duplicate of panel **(A)**, and data in panel **(M)** are duplicate of panel **(I)**. This was done to organize data in columns to simplify comparison.

According to the model, there should be no cross-coupling in the upright position, but cross-coupling to vertical would increase with the side-down tilt. Several typical examples of coding the spatial orientation of OKAN are shown in Figures 9–11. The VO neuron shown in Figure 9 received convergent inputs from the contralateral lateral and anterior canals as well as static otolith input (32). When the animal was tested in the upright position, firing rate of the unit decreased with optokinetic stimulation that induced OKN/OKAN to the left (Figure 9E). Firing rate increased for optokinetic stimulation that induced OKN/OKAN to the right (Figure 9A). When the OKN/OKAN to the left was induced with the animal tilted left side down (Figures 9B–D, V yaw), the yaw component of the OKAN became shorter with the tilt angle, while the cross-coupled downward vertical component of OKAN (V pitch) progressively increased with the tilt angle (Figures 9B–D). Similar changes were observed during OKN/OKAN to the right as he animal was tilted right side down (not shown). The same was true for OKAN to the right as the animal was tilted left side down (not shown) or right side down (Figures 9B–D, right side). In all cases, the changes in the neuronal firing rates were correlated only with changes of the yaw component of OKAN for both directions of rotation but not with the cross-coupled vertical component.

This unit was repeatedly tested in upright and side down tilts to obtain the relationship of neuronal firing rates to yaw and pitch OKN/OKAN (Figure 8C). The time constant of the yaw component of velocity storage, Tc, was largest when tested upright (two black and two gray symbols on the right in Figure 8C) and it progressively decreased with the tilt angle. The Tc of the decay in neuronal firing behaved similarly, and there was a tight linear relationship between the decrease in the Tc of the neuronal firing rate and the yaw component of OKAN. There was no difference for the data obtained for the slow phase eye velocities to the left or to the right (Figure 8C, black and gray symbols), which were associated with an increase and decrease in neuronal firing rate, except that the time constant of yaw OKAN to the right was slightly larger.

The linear regression coefficient remained approximately the same for left and right OKAN (1.27 ± 0.19) (Figure 8C). Similar responses were present for two other VO and three VPS neurons that received canal-related input only from the lateral canal or lateral and vertical canals (not shown). Thus, this type of neuron coded the horizontal component of velocity storage when there was cross-coupling to the pitch axis during OKN/OKAN. This relationship of firing rate to only yaw and not pitch OKN/OKAN indicates that the convergent input of contralateral anterior canal and otolith activity may have induced roll cross-coupling (see below), which was not tested for these units.

Thus, six (6/13) neurons including the neuron shown in Figure 9 coded the horizontal (yaw) component of OKN/OKAN in head coordinates.

One VPS neuron received convergent input from the contralateral posterior canal (Figure 10). With the animal upright, this unit decreased its fairing rate during OKN/OKAN to the left (Figures 10A,D) and increased it with OKN/OKAN to the right (not shown), regardless of the lack of the lateral canal input. When OKN/OKAN to the left was tested with the animal tilted left side down (Figures 10B,C), the firing rate of this neuron was clearly modulated in relationship to the cross-coupled upward pitch component of OKAN. There was no effect of head tilt to the right (Figures 10E,F) or tilt in either direction during OKN/OKAN to the right (not shown). This indicates that this neuron specifically coded the cross-coupled vertical component in a specific direction. Thus, neuronal changes in this experiment reflected activation of velocity storage. Six (6/13) neurons, including the neuron shown in Figure 10, coded the cross-coupled (pitch) component of OKAN. Furthermore, changes observed due to the induced cross-coupled component of OKAN in tilted position reflect spatial orientation of OKAN to gravity.

This indicates that VO and VPS neurons do not form a uniform category but form a distributed class of neurons that generate the velocity storage integrator and its output (Figure 1). This is consistent with previous findings for the one-dimensional analysis of neurons related to velocity storage (3).

It should be noted that six neurons as shown in Figure 9, had vertical canal-related inputs and yet changed their firing rate in relation to yaw, but not pitch OKAN. One of these neurons that received input from the ipsilateral anterior canal (32) was additionally tested during tilts forward and backward. Yaw OKN induced in these positions had only a yaw component, while OKAN cross-coupled to roll (Figure 11). When tested in the upright, activity of this unit slightly increased during OKN/OKAN to the left and decreased during OKN/OKAN to the right (Figures 11A,E,I,M). The relationship to the yaw component of OKN/OKAN remained the same when the animal was tilted up to 90° prone or right side down (not shown). When the animal was tilted left side down or supine, however, the changes in the firing rate became more evident and was associated with increased pitch and roll components (*y*), indicating a cross-coupled component from yaw (*z*). Thus, this neuron coded both of the cross-coupled components of velocity storage to roll and pitch. Specifically, cross-coupled upward pitch (Figures 11A–D) and CW from the animal’s point of view roll (Figures 11E–H) were associated with increases in the neuronal firing rate during OKN/OKAN to the left, while the cross-coupled downward pitch (Figures 11I–L) and CCW roll (Figures 11M–P) were associated with a decrease in the neuronal firing rate during OKN/OKAN to the right.

Racemic baclofen 6 mg (1.7 mg/kg) was injected IM and OKN/OKAN testing was repeated 30 min after injection. The slow phases of OKAN and changes in neuronal firing rate were completely eliminated, indicating that the velocity storage mechanism had been inactivated by the baclofen, and the firing rate of the neuron no longer had activity related to either the yaw or pitch components of the VOR (25). We did not have an opportunity to test the other five neurons for relations of their activity to cross-coupled roll OKAN. But, it is likely that at least some of them would have shown that relationship.

In summary, Figures 9–11 demonstrate that VO and VPS neurons code various aspects of the cross-coupled components of velocity storage. Six neurons (6/13, 46%) code only the horizontal component of velocity storage while cross-coupling occurs to the vertical plane. Six neurons code cross-coupled pitch and one coded the cross-coupled roll component of OKAN (7/13, 54%). Neurons shown in Figures 10 and 11 coded the cross-coupled vertical component of velocity storage in one (Figure 10, downward) or in both directions (Figure 11). Only one neuron, however, was tested for its relationship to the cross-coupled roll component of OKAN (Figure 11). Thus, the actual number of neurons that coded spatial orientation properties of velocity storage could be greater than 54%.

## Discussion

We characterized 33 VO and 11 vestibular-pause-saccade (VPS) neurons, recorded in the medial and SVN of alert cynomolgus monkeys. Using a newly developed technique, cells were studied for long periods during yaw rotation of the animals and visual surround while the monkeys were upright, prone and supine, and on-side.

Previous studies have shown the relationship of VO and VPS neurons to the time constant of velocity storage induced by angular rotation and OKN (4, 17). It was further demonstrated that velocity storage is implemented by cross connections between the rostral and MVN on the left and right sides (20, 21). The cross connections are GABA_b_-ergic (22–24) and are under control of the cerebellar nodulus, which is critical for controlling spatial orientation of velocity storage as well as its temporal properties (61–64). It is likely that VO neurons generate velocity storage, while VPS neurons are located in the path from the velocity storage integrator to the oculomotor plant, since their activity ceases during periods of drowsiness (3). The previous studies of velocity storage suggested that VO and VPS neurons should code all aspect of velocity storage including spatial orientation and habituation [see Ref. (15, 16, 25) for review]. However, due to technical limitations in recording of single neurons in three dimensions, a variety of tests that explore various aspects of velocity storage could not be done, limiting the understanding of how velocity storage is realized in three dimensions. The present study provides information that helps fill this gap.

All VO and VPS neurons had firing rates related to eye velocity during per- and post-rotatory nystagmus and OKAN. However, neuronal firing rates of the majority of tested neurons were tightly correlated with the horizontal component of velocity storage (Figure 8). Some of them (46%), however, were closely correlated with the horizontal component of velocity storage in head coordinates, regardless of head orientation in space (Figure 9). Other neurons were correlated with the cross-coupled components of velocity storage from yaw-to-pitch or yaw-to-roll (54%), during OKAN about tilted axes (Figures 10 and 11). Neuron shown in Figure 11 is similar to the other six neurons like the one shown in Figure 9. It had no relation to cross-coupled vertical component of OKAN. Firing rate of this neuron was clearly correlated to cross-coupled roll component of OKAN. Unfortunately, the other six neurons were not tested with animal in prone and supine positions. Thus, actual presence of neurons that coded velocity storage in spatial coordinates could be much higher than 54%. Together, the neurons, shown in Figures 10 and 11, code the horizontal component of eye velocity in spatial coordinates, as the head was reoriented from the spatial vertical. Thus, this study demonstrates that the majority of VO and VPS neurons are related to various aspects of the velocity storage mechanism. The time constants of a majority of the neurons were comparable to the time constant of velocity storage determined from ocular nystagmus (Figure 8).

Sixty-five percent of VO and VPS neurons in this study had asymmetrical responses for rotation to the left and to the right. The existence of such neurons is not unexpected, since the firing rates of many primary vestibular afferents increase to high levels during ipsilateral rotation but decrease to 0 during contralateral rotation, i.e., these primary afferents that, presumably, provide input to VO and VPS neurons have asymmetric responses (1).

The existence of central vestibular neurons sensitive to rotation in only one direction indicates that the velocity storage signals for rotation to the left and to the right are coded by different neurons. Further, these asymmetric neurons are clustered on opposite sides of the brainstem in the medial and SVN. These data indicate that the three-dimensional velocity storage integrator is composed of directional subsets of neurons that are likely the bases for the spatial characteristics of velocity storage. The majority of them receive input from the contralateral side. Thus, the interconnections between the neural subsets on either side are critical for the maintenance of this function, since velocity storage is completely lost when the axonal projections between the left and right medial and SVN are sectioned (20, 21).

Sinusoidal oscillations have typically been used to characterize the sensitivities of central vestibular neurons (65, 66). Our study indicates that sinusoidal rotation may not provide an accurate estimate of the characteristics of those neurons with asymmetric responses, especially those related to velocity storage. It has previously been shown that the sensitivity of VO and VPS neurons to head free gaze shifts cannot be predicted based on sinusoidal head-fixed oscillations (67, 68). Our study suggests that the reason may be due to the asymmetry of many VO and VPS neurons for rotations in alternate directions.

### How Neurons in This Study Fit to the Model of the Velocity Storage

This study demonstrates that VO and VPS neurons are not a uniform group of Type I and Type II neurons, consistent with earlier findings (3). Rather, the neurons recorded in this study consist of a variety of neural subgroups that implement the long time constant as well as the neurons that likely to implement the direct vestibular pathway that goes around velocity storage (Figure 1). The neurons that are involved in the realization of velocity storage probably project across the midline implementing a bilateral integrator, similar to that proposed for the velocity-position integrator (69, 70). Neurons that respond to rotation only in one direction also code velocity storage only for that direction. The bilateral model of velocity storage could be formed as two unilateral integrators connected to each other with cross-projections. The implementation of the velocity storage integrator in this fashion would be consistent with the abolition of velocity storage when the midline is cut (20, 21). This would also explain why the time constants of rotations in opposite directions are almost never identical (Figures 5A–C). It is also probable that the nodulus projects to these neurons (33), carrying otolith-related information (71) to implement the orientation properties of these velocity storage-related neurons (61, 63, 64).

We also demonstrated that some neurons coded velocity storage in head or canal coordinates. The firing rates of such neurons correlated with the horizontal (Figure 8) or the vertical (Figure 7) components of the VOR in one or both directions, but they were not related to the cross-coupled components of velocity storage. Such neurons could be involved in implementing the long time constant of the aVOR, but probably would not receive otolith information from the nodulus to be involved in spatial orientation of velocity storage. Moreover, it indicates that velocity storage may be implemented in canal coordinates and transformed to head coordinates within the vestibular nuclei. Thus, this study has demonstrated that the neural components in the vestibular nuclei have capability to not only implement the three-dimensional properties of velocity but also are part of the direct pathway around it as predicted by the model (Figure 1).

### Summary of Our Modeling Approach as Compared to Those of Others

There have been various models put forward that attempt to give different interpretations of velocity storage and the role it plays in vestibular processing (72–74) in opposition to the 3-D mechanistic velocity storage model presented by Raphan and Cohen (15, 16). One recent model seeks to put velocity storage in context of a Bayesian model of vestibular processing (74). In this framework, semicircular canal output drives an inverse model of semicircular canal dynamics, which attempts to reconstruct rotational velocity by integrating canal signals over time, but has a low-frequency cutoff to avoid accumulation of noise in the integrator. This is followed by a second internal model, which utilizes this reconstructed rotation velocity to compute an internal estimate of tilt and inertial acceleration. This internal model too is band-limited so as to limit the accumulation of drift in the estimate of tilt/translation over time. It is assumed that as a result of these two low-pass filters, low-frequency translation can be misinterpreted as tilt. These filters are conceptualized as two Bayesian priors of zero rotation velocity and zero linear acceleration, respectively (74) and velocity storage arises due to noise distribution of these Bayesian filters. However, regardless of how these filters are conceptualized (Bayesian or otherwise), these are global behavioral and perceptual models (75). Basing these filters on some obscure notion that they are there to separate tilt from translation (76–79) has not been related to neural activity and is not a credible approach to modeling the neural activity presented in this study. Moreover, it is beyond the scope of this study to examine and compare all models of velocity storage and how they are related to the data presented.

There are other more fundamental objections to the Bayesian tilt-translation-based model. An implication of the hypothesis that there is continual updating of the estimate of gravity in the Bayesian based model is not consistent with results obtained from centrifugation in the absence of gravity (80, 81). While in orbital space flight, gravity is imperceptible to the otoliths, and tilts of the linear acceleration vector only appear during centrifugation. Models, which depend on the continuous computation of gravity, and extracting tilt from translation based on Bayesian priors, would predict that during space flight there would be no perception of tilt or OCR during centrifugation, only perception of translation and compensatory eye movements in response to this translation. This idea had been formalized as the otolith tilt-translation reinterpretation hypothesis (82, 83). Results from flight experiments are contrary to these predictions. Centrifugation during space flight induced a clear perception of tilt (80). It also produced OCR, which was the same as that induced by similar linear acceleration on earth during static tilt (81).

A simpler explanation for the maintenance of perception and ocular tilt in the absence of gravity is that instantaneous tilt of the head is determined by a computation of head orientation relative to the net GIA, which is used as the spatial vertical reference (80, 81). Thus, there may be no need for continuous updating of the acceleration of gravity relative to the head on earth, as implied by the Observer or Bayesian based models (72, 74, 78). Orientation could simply be determined mechanistically by a three-dimensional filtering mechanism, which automatically determines the “head vertical re GIA estimate” through the eigenvectors of the system matrix, possibly controlled by the cerebellum (84–86). The neural recordings in this study and its direct relationship to the model support this hypothesis.

In contrast to the Bayesian and internal models that have shown no direct link to central vestibular neural activity, we have previously demonstrated a direct link of our model to neural activity in the central vestibular system in a number of studies. We have demonstrated that otolith polarization vectors of VO neurons can be adapted and that the polarization vector of VO neurons adapt toward the axis of gravity if animals are positioned away from spatial vertical (29, 49). Interestingly, EHV and PVP neurons (87) as well as central otolith neurons (45) have a very limited adaptive capability of their polarization vectors. The present study for the first time has related unit activity recorded in the central vestibular system to the mechanistic three-dimensional model of velocity storage presented by Raphan and Cohen (12, 14, 16). We, therefore, reject the notion that velocity storage is an outcome of noise in differentiating tilt from translation computations (74). Moreover, we do not see how such a notion can be reflected in the central VO neurons presented in this paper. In contrast, the head vertical and its relationship to the spatial vertical are features of the Raphan–Cohen model and is reflected in the eigenvalues and eigenvectors of the system matrix, H (Figure 1), and is a function of differential otolith activation (12, 14).

### Some Clinical Implications of Velocity Storage

The time constant of vestibular nystagmus is an important measure when evaluating vestibular abnormalities (88, 89). Typically, the time constant of velocity storage is reduced with unilateral and bilateral vestibular lesions. It can also be habituated by repeated rotations (90, 91). Such reduction reduces the susceptibility to motion sickness (25, 92, 93). This shows that velocity storage is not only critical for spatial orientation with regard to gravity, but it also serves as an input to the sympathetic system, and motion sickness susceptibility can be reduced by shortening the VOR (velocity storage) time constant (94). Studies in monkeys also demonstrate that prolonged oscillation in roll while rotating about a spatial vertical axis induces oscillatory modulations of nystagmus (95) similar to those in patients with the Mal de Debarquement Syndrome (MdDS) (94, 96, 97). Thus, changes in velocity storage are postulated to be responsible for the postural instability induced by prolong travel on water (96). Of particular significance is the fact that the MdDS impacts the body postural system, resulting in rocking, or swaying at 0.2 Hz, showing that the velocity storage integrator not only is associated with spatial orientation, eye movements and activation of the sympathetic system, but also with descending vestibulo-spinal projections that are associated with strong postural instability during the MdDS. While data in this study were obtained from monkeys they are still directly applicable to humans. The works of Jell and colleagues (98, 99) and from our laboratory (100) have shown that the observed time constant is dependent on the size and quality of the stimulus. Moreover, even when the stimulus is such that the time constants are small, the cross-coupling, which depends on the relationship of the roll, pitch, and yaw time constants, is still maintained (101). Thus, further shortening of the yaw time constant or adapting its orientation clearly reduces susceptibility to motion sickness in that rolling the head at the frequency of rocking/swaying in MdDS while velocity storage is activated by OKN significantly reduce MdDS symptoms.

## Conclusion

This study identifies a mechanism that is widely distributed among vertebrate species that converts the activity in semicircular canal and otolith organ afferents to code angular velocity of the head in three dimensions. Located in the medial and SVN, it has an additional component, particularly evident in primates and some mammals, namely a mechanism that orients the axis of eye rotation and probably balance to the spatial vertical. The data in this report provide the first three-dimensional study of the neurons that underlie this function. There is no reason to hypothesize that the velocity storage mechanism is involved in separating tilt from translation. There are no specific neural recordings to date that have related neural mechanisms to velocity storage and the relation of velocity storage to the tilt-translation hypothesis is unsubstantiated speculation (74, 102, 103). The data presented here is the first of its kind to clearly show neural recordings that are related to a mechanistic three-dimensional model of velocity storage [(12, 14); see Ref. (16) for review]. It is critical now to determine how orientation is controlled by the vestibulocerebellum to determine the fundamental aspect of balance, which is simply at best, is body orientation to the spatial vertical. In support of this idea, our previous findings indicate that orientation of otolith convergent input to VO and vestibular-pause-saccade (VPS) neurons could be adapted by prolong head side-down orientation (29, 87). Such plasticity suggests the possibility of development of new techniques to enhance movement and orientation in three-dimensional space.

## Ethics Statement

The surgical procedures and experimental protocol conformed to the Guide for the Care and Use of Laboratory Animals and were approved by the Institutional Animal Care and Use Committee of Icahn School of Medicine at Mount Sinai.

## Author Contributions

SY: planning experiments, collection of all data, data processing, data analyses, figure making, and writing of this manuscript. TR: planning experiments, data analyses, figure making, and writing of this manuscript. BC: planning experiments, figure making, and writing of this manuscript.

## Conflict of Interest Statement

The authors declare that the research was conducted in the absence of any commercial or financial relationships that could be construed as a potential conflict of interest.

## Abbreviations

VOR, angular vestibulo–ocular reflex; MVN, medial vestibular nucleus; SVN, superior vestibular nucleus; VO, vestibular-only neuron in medial vestibular nucleus; VPS, vestibular-pause-saccade neuron in medial vestibular nucleus.
